# Beyond human expertise: the promise and limitations of ChatGPT in suicide risk assessment

**DOI:** 10.3389/fpsyt.2023.1213141

**Published:** 2023-08-01

**Authors:** Zohar Elyoseph, Inbar Levkovich

**Affiliations:** ^1^Department of Psychology and Educational Counseling, The Center for Psychobiological Research, Max Stern Yezreel Valley College, Emek Yezreel, Israel; ^2^Department of Brain Sciences, Faculty of Medicine, Imperial College London, London, United Kingdom; ^3^Faculty of Graduate Studies, Oranim Academic College, Kiryat Tiv'on, Israel

**Keywords:** artificial intelligence, ChatGPT, diagnosis, psychological assessment, suicide risk, risk assessment, text vignette

## Abstract

ChatGPT, an artificial intelligence language model developed by OpenAI, holds the potential for contributing to the field of mental health. Nevertheless, although ChatGPT theoretically shows promise, its clinical abilities in suicide prevention, a significant mental health concern, have yet to be demonstrated. To address this knowledge gap, this study aims to compare ChatGPT’s assessments of mental health indicators to those of mental health professionals in a hypothetical case study that focuses on suicide risk assessment. Specifically, ChatGPT was asked to evaluate a text vignette describing a hypothetical patient with varying levels of perceived burdensomeness and thwarted belongingness. The ChatGPT assessments were compared to the norms of mental health professionals. The results indicated that ChatGPT rated the risk of suicide attempts lower than did the mental health professionals in all conditions. Furthermore, ChatGPT rated mental resilience lower than the norms in most conditions. These results imply that gatekeepers, patients or even mental health professionals who rely on ChatGPT for evaluating suicidal risk or as a complementary tool to improve decision-making may receive an inaccurate assessment that underestimates the actual suicide risk.

## Introduction

ChatGPT is a tool developed by OpenAI that is based on GPT language model technology and available in the public domain (1). In the few months since it was launched on 30 November 2022, ChatGPT has gained a remarkable 100 million users, making it the most rapidly growing consumer application to date (2). ChatGPT is a highly sophisticated chatbot that can handle text-based requests ranging from simple queries to more advanced tasks. It comprehends and interprets user requests and generates appropriate responses in nearly natural human language (3). This ability to generate human-like language and perform complex tasks makes ChatGPT a significant breakthrough in the fields of natural language processing and artificial intelligence (2, 3). Most studies conducted on ChatGPT have focused on its use in academia (4), whereas exploration of its applications in the field of applied psychology has been limited. While ChatGPT demonstrates promising theoretical potential (5), its clinical capabilities in the field of mental health remain unclear, and particularly its ability to address critical issues such as suicide prevention, a significant mental health concern.

Suicide constitutes a major health problem and cause of death across the globe. Of the approximately 90–100 suicide attempts per 100,000 individuals each year in Israel, 7.9 result in death (6, 7). In the United States, the death rate is 14.2 per 100,000 suicide attempts (8). Psychiatric diseases are at least 10 times more prevalent among individuals who attempt and/or commit suicide than in the general population (9). Suicide attempts are often impulsive and occur at times of crisis as a response to being unable to manage daily stresses and demands (10). Among patients discharged from the hospital following a suicide attempt, the risk of a subsequent attempt during the first 3 years after hospitalization is 12%–30% (11).

In response, considerable efforts have been invested in developing effective suicide prevention strategies to reduce the risk of recurring attempts (12). Early identification of individuals at risk of suicide is a fundamental prevention strategy, particularly among the high-risk population of mental health patients (13). Hence, clinicians’ ability to recognize indicators of suicide potential is essential to implement appropriate crisis management and suicide intervention strategies, especially during times of acute crisis (14). Given the magnitude of the problem, significant investment in suicide prevention programs is crucial. Recent initiatives have focused on training gatekeepers from various community groups (e.g., teachers, policymakers, and military commanders) to detect indicators of suicide risk (15, 16). These efforts aim to broaden the pool of professionals capable of evaluating suicide risk beyond psychiatrists and clinical psychologists working in hospital settings. The term “gatekeeper” refers to individuals who are in direct contact with those who may be at risk of suicide and can be trained to identify significant suicide risk factors. Gatekeeper training programs are often part of broader suicide prevention initiatives aimed at enhancing knowledge and providing training for identifying risk factors and suicidal behaviors. These programs also aim to equip participants with the necessary skills to assess an individual’s level of risk and to manage the situation effectively by providing access to appropriate resources and treatment referrals (17). The role of gatekeepers is to facilitate access to appropriate care and treatment for those at risk of suicide (17). Research suggests that such gatekeepers can be useful in reducing suicide as part of a systematic approach to suicide prevention (18–20). In theory, artificial intelligence (AI) has the potential to support gatekeepers in their decision-making processes and improve the effectiveness of formal psychometric tools and clinical assessments in predicting suicide behavior. Currently, these methods are often found to have insufficient predictive capabilities (21, 22).

### The current study

In the current research, we investigated the identifiable limitations of ChatGPT to evaluate suicide risk and to identify associated factors. In addition, we examined whether suicidality risk assessment contains fundamental principles of the Interpersonal Theory of Suicide (ITS), a well-established and empirically supported theoretical framework proposed by Joiner (23, 24) for assessing the risk of suicide and identifying associated factors. Specifically, we examined how the two core dimensions of ITS, namely *perceived burdensomeness* and *perceived thwarted belongingness*, influence therapists’ perceptions and evaluations of suicide ideation and behavior. The term *perceived burdensomeness* refers to a psychological state in which individuals perceives that their very existence imposes a burden on their family, friends, and/or society. According to Joiner et al. (25), this perception leads individuals to believe that their death would be more beneficial than their continued life, in terms of the impact on their family, friends, society, and so on. As defined by Van Orden et al. (24), the concept of *thwarted belongingness* refers to a feeling of estrangement from others, with particular emphasis on the distressing sensation of being excluded from one’s family, friends, or other cherished groups. This construct appears to play a crucial role in suicide, as it represents a basic psychological requirement. Failure to meet this need may lead to an inescapable sense of distress (26). We specifically selected perceived *burdensomeness* and *thwarted belongingness*, excluding other risk factors like gender, age, access to weapons, etc., because they provide a closer reflection of the patient’s mental experience. By adopting this approach, we aim to investigate ChatGPT’s identifiable limitations to assess suicidal risk based on how the patient interprets their interpersonal experiences.

The objective of the current study:

To exploring identifiable limitations using ChatGPT as a suicide risk assessment tool across two identifiable variables perceived burdensomeness and thwarted belongingness.

## Methods

### Artificial intelligence procedure

Using the free version of ChatGPT (OpenAI San Francisco), we conducted three evaluations during the period of 20–30 March 2023 (ChatGPT March 14 version) to examine how perceived burdensomeness and thwarted belongingness affect the performance of ChatGPT in assessing various aspects of suicidal risk compared to the performance of human therapists.

### Input source

In the present investigation, we adopted the text vignette methodology as elucidated by Bresin et al. (27) to examine the observer’s perspective of suicidal acts. A specific vignette from Levi-Belz and Gamliel (28) was entered to the ChatGPT interface. The chosen vignette contains general background and two additional paragraphs: one for evaluating low or high perceived burdensomeness and the other for assessing low or high thwarted belongingness.

#### General background

*Jane is a 37-year-old single woman without children, currently living alone, but in her past spent a long time living with her father, who passed away 2 years ago following a severe illness. Jane holds a B.A. degree and has kept a steady job for 10 years. Recently, however, her manager informed her that, due to financial cuts, she must dismiss her. Jane had a close, intimate relationship for the past year with a boyfriend, her first serious and meaningful one; she was very excited about the relationship, hoping it would lead to marriage. However, following a period of repeated quarrels, her boyfriend informed her he no longer loved her, and he left her. Jane was left very sad and hurt. In recent weeks she keeps thinking about the relationship over and over again, trying to analyze what happened. Jane is experiencing a heavy emotional load, she is very tired, lacking energy, and finding it hard to sleep nights. Her routine tasks have become difficult for her, and she finds it hard to concentrate and to function properly. She dreads the future, and it seems to her that there is no chance that she would find a love like that again.*

#### Low perceived burdensomeness condition

*In general, Jane feels she has abilities. She feels she has something to contribute to the world and to others around her, and sometimes people come to her for help and advice. She feels important and meaningful, and she seems to believe in herself.*

#### High perceived burdensomeness condition

*Jane feels worthless. Often she experiences herself as a burden to the people around her and she feels that it would be better if she were gone. She often sees herself as having no real meaning in other people’s lives and she experiences feelings of self-hatred. For example, she is angry at herself and thinks that the departure of her boyfriend was her fault.*

#### Low thwarted belongingness condition

*Jane has some close friends, and she feels that she can tell them about what she has been going through in recent weeks. In general she makes connections relatively easily and, despite the recent crisis, she feels lucky because there are people around who care for her.*

#### High thwarted belongingness condition

*Jane is experiencing significant loneliness in general and she has only some, if any, close friends around her. She is very withdrawn, distant from others, and she feels that there are no people to whom she could turn in times of trouble. Therefore, she has not shared her feelings with anyone since the breakup.*

### Measures

After each of the four conditions *(1. low burdensomeness and low belongingness; 2. low burdensomeness and high belongingness; 3. high burdensomeness and low belongingness; 4. high burdensomeness and high belongingness),* we submitted six questions to ChatGTP taken from Levi-Belz and Gamliel (28). Each question was categorized into one of four subscales: (1) Psychache (i.e., psychological pain) (Q1: *“What do you think is Jane’s level of distress?”*; Q2: *“What do you think is Jane’s level of mental pain?”*); (2) Suicidal ideation (Q3: *“What is the likelihood Jane will have suicidal thoughts?”*); (3) Risk of suicide attempt (Q4: *“What is the likelihood Jane will attempt suicide?”*; Q5: *“What is the likelihood Jane will make a serious suicide attempt?”*); and (4) Resilience (Q6: *“How do you assess Jane’s level of resilience?”*). All Six questions employed a seven-point Likert type scale, estimating intensity (Q1, Q2, Q6) and likelihood (Q3–Q5), ranging from 1 (very slight) to 7 (very high).

### Scoring

ChatGPT’s performance was scored according to Levi-Belz and Gamliel (28). We then compared the ChatGPT scores to the norms of 379 participants trained in mental health professions (21% male, mean age 36 ± 8.8), 53 participants who were psychology graduate students, 266 with a master’s degree, and 60 with a doctorate. In terms of professional roles, 43 participants held certifications as supervisors in their mental health specialty, 108 were certified experts, 128 were interns, and 100 either had not begun their internship or were in professions not mandating an internship. The majority of the sample, accounting for 84%, comprised practicing mental health professionals, while the remaining individuals had previous experience in the mental health field but were currently inactive (28).

### Statistical analysis

The data were presented as means ± SDs and as percentages of the first second and third evaluations. Two-sample *t*-tests were used to evaluate the differences between the average ChatGPT performance on the three evaluations and the norms of the mental health professionals reported by Levi-Belz and Gamliel (28).

## Results

Table 1 depicts ChatGPT’s performance (mean ± SD) for all four conditions (1. low burdensomeness and low belongingness; 2. low burdensomeness and high belongingness; 3. high burdensomeness and low belongingness; 4. high burdensomeness and high belongingness) for the four dependent variables (1. psychache; 2. suicidal ideation; 3. risk of suicide attempt, and 4. resilience) compared to the norms of the health professionals reported by Levi-Belz and Gamliel (28).

**Table 1:** Descriptive statistics of the four variables—psychache, suicidal ideation, risk of suicide attempt, and resilience—as a function of perceived burdensomeness and perceived thwarted belongingness.

Thwarted belongingness	Perceived burdensomeness	Psychache				Suicidal ideation			
		MHP	ChatGPT		MHP vs. ChatGPT	MHP	ChatGPT		MHP vs. ChatGPT
		Mean ± SD	Mean ± SD	%		Mean ± SD	Mean ± SD	%	
Low	Low	5.48 (0.75)	6.00 (0)	75.6	*t* = −6.3^***^	3.60 (1.28)	4.00 (0)	62.2	*t* = −2.88^**^
	High	5.91 (0.72)	6.00 (0)	55.0	ns	4.98 (1.29)	4.00 (0)	22.4	*t* = 7.0^***^
High	Low	5.87 (0.64)	6.00 (0)	58.0	ns	4.29 (1.36)	4.00 (0)	41.6	ns
	High	6.15 (0.65)	6.33 (0.24)	61.1	ns	5.37 (1.11)	5.33 (0.47)	48.7	ns
Thwarted belongingness	Perceived burdensomeness	Risk of suicide attempt				Resilience			
		MHP	ChatGPT		MHP vs. ChatGPT	MHP	ChatGPT		MHP vs. ChatGPT
		Mean ± SD	Mean ± SD	%		Mean ± SD	Mean ± SD	%	
Low	Low	2.27 (1.03)	1.50 (0)	22.0	*t* = 6.89^***^	5.12 (0.84)	3.66 (0.47)	4.2	*t *= 5.06^*^
	High	3.12 (1.21)	1.50 (0)	9.0	*t* = 12.34^***^	4.49 (0.99)	3.00 (0)	6.6	*t* = 13.87^***^
High	Low	2.92 (1.26)	1.50 (0)	13.0	*t* = 10.39^***^	4.20 (0.97)	3.33 (0.47)	18.6	ns
	High	4.08 (1.18)	2.70 (0.47)	5.2	*t* = 6.36^***^	3.44 (1.16)	3.00 (0)	35.2	*t* = 3.49^***^

MHP, Mental Health Professionals; ^*^0.05, ^**^0.01, ^***^0.001.

### Psychache

In the low burdensomeness and low belongingness condition, ChatGPT assessed the level of psychache as higher than the sample of mental health professionals (75th percentile, *t* = −6.3, *p* < 0.001). In the other conditions, no significant differences were found (percentile range 55–61). The ChatGPT mean scores and standard deviations were the same (6 ± 0) in the first three conditions.

### Suicidal ideation

**Figure 1:**
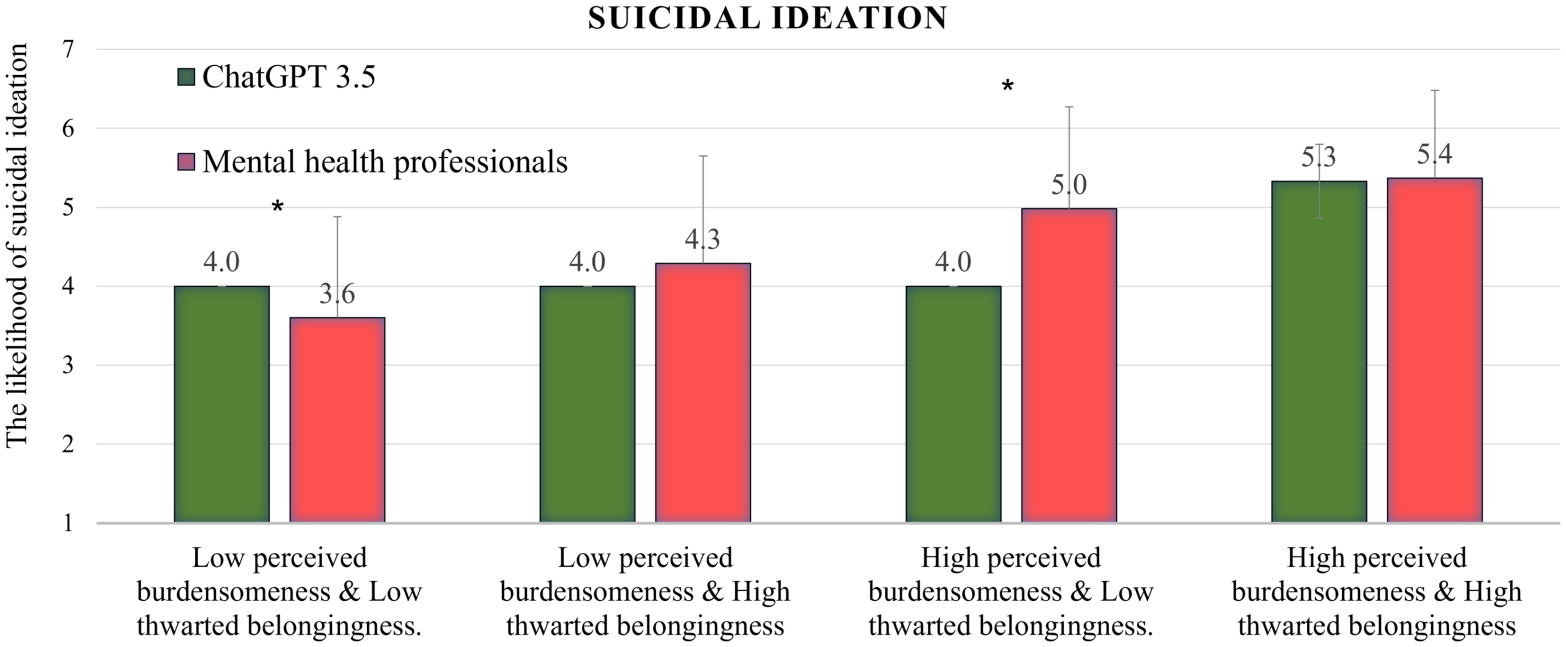
ChatGPT’s performance in all four conditions on the suicidal ideation variable, compared to the norms of mental health professionals; *p < 0.01.

As can be seen in Figure 1, in the low burdensomeness and low belongingness condition, ChatGPT assessed the level of suicidal ideation as higher than the sample of mental health professionals (62nd percentile, *t* = −2.8, *p* < 0.01). In the low burdensomeness and high belongingness condition, ChatGPT assessed the level of suicidal ideation as low compared to the mental health professionals sample (22nd percentile, *t* = 7.00, *p* < 0.001). In the other two conditions, no significant differences were found (percentile range 41–48). The ChatGPT mean scores and standard deviations were the same (4 ± 0) in the first three conditions.

### Risk of suicide attempt

**Figure 2:**
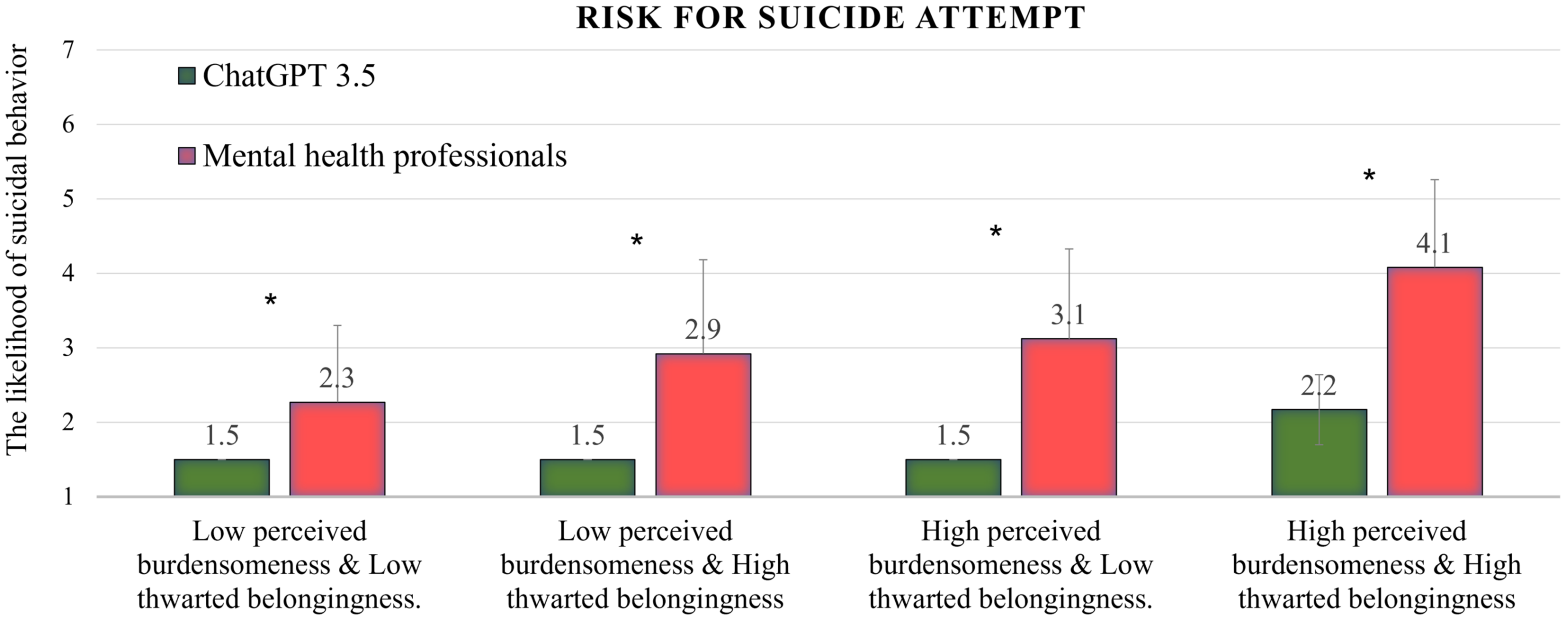
ChatGPT’s performance in all four conditions on the risk for suicide attempt variable, compared to the norms of mental health professionals; *p < 0.001.

Figure 2 shows that in all four conditions, ChatGPT assessed the level of risk of suicide attempts significantly lower than did the mental health professionals sample (percentile range 5–22; *t*-test range = 6.36–12.34; *p* < 0.001 for all conditions). In the condition reflecting the highest level of risk (high burdensomeness and high belongingness), the ChatGPT assessment was ranked in the lowest percentile (5th). The ChatGPT mean scores and standard deviations were the same (1.5 ± 0) in the first three conditions.

### Resilience

In all conditions except the low burdensomeness and high belongingness condition, ChatGPT assessed the level of resilience as significantly lower compared to the mental health professionals sample (percentile range 4–35; *t*-test range = 3.49–13.87; *p* < 0.001 for low burdensomeness and high belongingness and high burdensomeness and high belongingness, *p* < 0.05 for low burdensomeness and low belongingness). In the low burdensomeness and high belongingness conditions, no significant differences were found (18th percentile).

## Discussion

This study aimed to examine exploring identifiable limitations of ChatGPT evaluates suicidal behavior and contributing factors in comparison to the norms established by Levi-Belz and Gamliel (28) for healthcare professionals. This study offers a unique contribution by evaluating ChatGPT ability to assess suicidal risk in the context of perceived burdensomeness and thwarted belongingness. To the best of our knowledge, this issue has not been explored in previous research.

The findings show that ChatGPT consistently underestimated the likelihood of suicide attempts across all scenarios when compared to mental health professionals. Specifically, in the condition involving high burdensomeness and high belongingness, which has the highest risk level according to the Interpersonal Theory of Suicide (ITS), ChatGPT ranked the risk in the lowest percentile (5th). The absolute value of suicidal behavior was greater in this condition than in the other three, suggesting that ChatGPT takes perceived burdensomeness and thwarted belongingness into account to some extent, albeit to a lesser degree than what is suggested by theory and clinical experience (23, 24, 28). While the assessment of mental health professionals was influenced by the presence of either perceived burdensomeness or thwarted belongingness, ChatGPT’s assessment was only affected when both factors were present, and even then, only to a minimal extent.

In most conditions, ChatGPT tended to rate mental resilience lower than the norms. In addition, the psychache and suicidal ideation variables were pretty similar to the norms in most of the conditions. These results indicate that ChatGPT’s assessment of risk of suicide attempt is less influenced by factors such as resilience (28–30). Based on the findings, we can deduce that ChatGPT’s overall evaluation might not fully encompass the complexities of assessing suicidal risk, and its capacity to identify potential risk and protective factors may deviate from established clinical expertise. While chatbots show promise in improving the insufficient predictive capability of clinical assessments and suicide risk questionnaires (21, 22), it is evident that, within the context of this study, they are unable to effectively fulfill this role.

These results differ from those of contemporary artificial intelligence (AI) models for evaluating suicidal risk (31, 32). Consequently, the study emphasizes the importance of exercising prudence and further refining AI models for appraising suicidal risk. The purpose of this study was to explore the potential of artificial intelligence (AI) in evaluating mental health risks beyond its theoretical proficiency and semantic knowledge, as discussed in previous literature (33, 34). Specifically, ChatGPT shows major potential for contributing to assessment processes in mental health (35), among other things due to its vast knowledge, high accessibility on any phone or computer, and ability to reduce the feeling of stigma and shame associated with psychological or psychiatric settings. Previous research on the application of AI in mental health has focused primarily on its potential to aid in technical tasks, thereby reducing the need for clinical interactions. This approach suggests that AI technologies can assist in completing non-personalized tasks, thus freeing up clinicians’ time to focus on delivering more empathic care and “humanizing” their practice, as the quality of mental health care often relies on the clinician-patient relationship (36). Scholars have proposed several potential applications of AI in the mental health field, including assisting clinicians with time-consuming tasks such as updating medical records, improving diagnostic and prognostic accuracy, promoting understanding of mental illnesses mechanisms, and enhancing treatment based on biological feedback (37–40). The findings of the current study suggest that using ChatGPT to evaluate suicide risk through vignettes or as a tool for improved decision-making among clinicians or gatekeepers is not in line with clinical experience and is not recommended. The implication is that ChatGPT should be used to direct individuals who pose questions about suicide to receive mental health treatment or assistance.

The present study has several limitations. First, it focused on the March 13 version of ChatGPT. More recent versions have since been released, and forthcoming studies should investigate this issue using these updates. Second, the study focused on a vignette featuring a female participant, whereas the incidence of suicide deaths is higher among males. Therefore, we recommend conducting additional studies to investigate vignettes featuring various demographic groups, including male participants, psychiatric patients, adolescents, and older individuals. Third, we compared the ChatGPT data with a sample of data from mental health professionals in Israel. Therefore, we recommend investigating the appraisal of therapists from other countries to assess cross-cultural differences. Fourth limitation is that the research on which we were based (28) did not report the degree of reliability between the judges. Accordingly, an assessment of the degree of variability in human responses can be roughly estimated through standard deviations alone. Lastly, This research delved into the intricate realm of suicide risk assessment using artificial intelligence. However, in order to establish a more expansive understanding, further studies are necessary. These subsequent investigations should explore supplementary risk factors, incorporate additional large language models, analyze data at various time points, and compare findings with a wider range of clinical samples.

## Conclusion

The objective of this research was to investigate the extent of ChatGPT’s ability to evaluate suicide risk and to identify few factors compared to the assessment of mental health professionals and to explore how this assessment is influenced by perceived burdensomeness and thwarted belongingness. The findings revealed that in all scenarios ChatGPT consistently assessed the risk of suicide attempt as lower than did mental health professionals. The results of this study imply that ChatGPT’s evaluation of suicidal ideation and suicide attempt risk may be less affected by elements such as resilience, perceived burdensomeness, and thwarted belongingness. The finding that ChatGPT underestimates the risk of suicide attempts in comparison to mental health care professionals, especially in the most severe condition, is a significant cause for concern. It implies that gatekeepers, patients, or even mental health professionals who are considering using ChatGPT for evaluating suicidal risk may receive an inaccurate assessment that underestimates the actual risk. Despite the theoretical possibility that ChatGPT’s assessments are more precise, it is incumbent upon mental health professionals to prioritize human life and treat the chat assessments in this domain as unprofessional until further evidence becomes available.

## Data availability statement

The raw data supporting the conclusions of this article will be made available by the authors, without undue reservation.

## Author contributions

ZE: conception and design of the study, acquisition and analysis of data, and drafting of a significant portion of the manuscript and figures. IL: conception and design of the study, acquisition and analysis of data, and drafting of a significant portion of the manuscript. All authors contributed to the article and approved the submitted version.

## Funding

This research was supported by Oranim College (no. 5100260).

## Conflict of interest

The authors declare that the research was conducted in the absence of any commercial or financial relationships that could be construed as a potential conflict of interest.

## Publisher’s note

All claims expressed in this article are solely those of the authors and do not necessarily represent those of their affiliated organizations, or those of the publisher, the editors and the reviewers. Any product that may be evaluated in this article, or claim that may be made by its manufacturer, is not guaranteed or endorsed by the publisher.
